# Biodistribution and PET Imaging of pharmacokinetics of manganese in mice using Manganese-52

**DOI:** 10.1371/journal.pone.0174351

**Published:** 2017-03-17

**Authors:** A. Lake Wooten, Tolulope A. Aweda, Benjamin C. Lewis, Rebecca B. Gross, Suzanne E. Lapi

**Affiliations:** 1 Mallinckrodt Institute of Radiology, Washington University School of Medicine, St. Louis, Missouri, United States of America; 2 Department of Biomedical Engineering, Washington University, St. Louis, United States of America; 3 Department of Physics, Washington University, St. Louis, United States of America; 4 Department of Radiology, University of Alabama at Birmingham, Birmingham, United States of America; Maastricht University Medical Centre, NETHERLANDS

## Abstract

Manganese is essential to life, and humans typically absorb sufficient quantities of this element from a normal healthy diet; however, chronic, elevated ingestion or inhalation of manganese can be neurotoxic, potentially leading to *manganism*. Although imaging of large amounts of accumulated Mn(II) is possible by MRI, quantitative measurement of the biodistribution of manganese, particularly at the trace level, can be challenging. In this study, we produced the positron-emitting radionuclide ^52^Mn (*t*_*1/2*_ = 5.6 d) by proton bombardment (*E*_*p*_<15 MeV) of chromium metal, followed by solid-phase isolation by cation-exchange chromatography. An aqueous solution of [^52^Mn]MnCl_2_ was nebulized into a closed chamber with openings through which mice inhaled the aerosol, and a separate cohort of mice received intravenous (IV) injections of [^52^Mn]MnCl_2_. *Ex vivo* biodistribution was performed at 1 h and 1 d post-injection/inhalation (p.i.). In both trials, we observed uptake in lungs and thyroid at 1 d p.i. Manganese is known to cross the blood-brain barrier, as confirmed in our studies following IV injection (0.86%ID/g, 1 d p.i.) and following inhalation of aerosol, (0.31%ID/g, 1 d p.i.). Uptake in salivary gland and pancreas were observed at 1 d p.i. (0.5 and 0.8%ID/g), but to a much greater degree from IV injection (6.8 and 10%ID/g). In a separate study, mice received IV injection of an imaging dose of [^52^Mn]MnCl_2_, followed by *in vivo* imaging by positron emission tomography (PET) and *ex vivo* biodistribution. The results from this study supported many of the results from the biodistribution-only studies. In this work, we have confirmed results in the literature and contributed new results for the biodistribution of inhaled radiomanganese for several organs. Our results could serve as supporting information for environmental and occupational regulations, for designing PET studies utilizing ^52^Mn, and/or for predicting the biodistribution of manganese-based MR contrast agents.

## Introduction

Manganese is a micronutrient that is essential for human life in only trace amounts [1, 2], but deficiency is rare [3]. Existing primarily in the Mn(IV) oxidation state in the earth’s crust [4], manganese is the third most abundant transition metal in the crust [4] and is taken up by plants, where it is utilized in green leaves as an oxidizer in photosynthesis [4]. Manganese(II) is by far the most electrochemically stable oxidation state for manganese [4] and the state that is predominantly used as an enzymatic cofactor. Manganese in the blood is found as Mn(III) bound to binding sites for Fe(III) on transferrin [5, 6] or as Mn(II) bound to other serum proteins [7], but the vast majority of manganese leaves the blood quickly and is accumulated in various organs or excreted [8]. The necessity of manganese as a nutrient is sometimes overshadowed by its neurotoxic effects that can result in *manganism* [2], a condition that was first described in 1837 [9]. In mangansim, early-stage patients often present with psychiatric symptoms [10, 11] that are sometimes referred to as “manganese madness” [12]. Later-stage patients develop symptoms of parkinsonism, including intellectual impairment, tremors, and rigidity similar to idiopathic Parkinson’s disease [9, 12–14], with a few key differences [12].

In addition to studying the neurotoxicity of manganese, an additional motivation for understanding the biodistribution of free manganese is that Mn(II) can be utilized in contrast agents for manganese-enhanced magnetic resonance imaging (MEMRI), which was studied early in the history of MRI by P. Lauterbur and colleagues [15] and later achieved *in vivo* [16]. Manganese(II) is “high-spin” from its five unpaired valence electrons, which shorten the spin-lattice time constant (*T*_*1*_) for nearby ^1^H nuclei, resulting in brighter signal (when using a *T*_*1*_-weighted MR pulse sequence) [8, 17–19]. In fact, *T*_*1*_-weighted MRI may be used as an indicator of manganese-induced parkinsonism [20, 21]. Additionally, chelated Mn(II) could serve as an alternative to Gd(III), which is currently the most commonly used cation core in *T*_*1*_-shortening contrast agents for MRI, since free Gd(III) is excreted through the kidneys, but free Mn(II) is excreted almost exclusively in feces, primarily from bile from the liver [22–25].

Thus far, the only manganese-based MR contrast agent to reach the market in the United States was Teslascan® (*mangafodipir*, manganese(II)-dipyridoxyl diphosphate, Mn-DPDP) (Amersham plc, Amersham, Buckinghamshire, United Kingdom), a chelated form of Mn(II) that reduces cardiac toxicity, by slowing the availability of free Mn(II) [17, 26–28] due to dissociation [29]. Mn-DPDP was officially indicated for detecting liver metastases of colorectal cancer [26, 27], but it no longer on the market in the United States. Various other chelated forms of Mn(II) and Mn(III) [30] are being developed, numerous kinds of Mn-doped inorganic and organic nanoparticles are in the *in vitro* and pre-clinical stages [19], and oral administration of liquids containing Mn(II) has even been tested in humans [31, 32]. However, since MRI is not inherently as quantitative as PET, it is valuable to develop surrogates that are radiolabeled with a positron-emitting radioisotope of manganese [27, 28, 33, 34] for quantitative confirmation of their biodistribution.

Although imaging of large amounts of accumulated Mn(II) is possible by *T*_*1*_-weighted MRI, true quantification of the biodistribution of non-radioactive manganese *in vivo* is difficult. However, pre-clinical imaging and *ex vivo* biodistribution using tracer amounts of radiomanganese can provide a route to quantify the amount of manganese as a percentage of administered dose in animal models. Past studies have examined the biodistribution of manganese in various forms administered by various routes, mostly in rodents, and have generally found that manganese is distributed to many tissues throughout the body. Several of these studies have used the radiotracers ^52^Mn (*t*_*1/2*_ = 5.6 d [35]), and ^54^Mn (*t*_*1/2*_ = 312 d [35]), or by administering stable manganese (^55^Mn: 100% natural abundance [35]) followed by various techniques for quantifying non-radioactive metals in the tissues [17, 36–38]. More recently, researchers at the University of Wisconsin have published results for *ex vivo* biodistribution of IV ^52^Mn(II) in rodents: Graves, et al. [39] have investigated biodistribution in tumor-bearing mice at 96 h post-injection (p.i.), as well as by *in vivo* PET at timepoints 4–96 h p.i. Additionally, Brunnquell, et al. [40] have investigated biodistribution in rats at 4 and 48 h p.i.

The half-life of ^52^Mn is long enough for *in vivo* imaging at timepoints as long as days or even weeks p.i., and ^52^Mn emits positrons at a sufficient branching ratio (*I*_*β*+_ = 29.6% [35]) for *in vivo* medical imaging by positron emission tomography (PET). Manganese-52 provides the additional benefit of low-energy positron emission (*E*_*β+*_ = 242 keV [35]) (resulting in better spatial resolution in PET). Manganese-52 can be produced efficiently by the ^52^Cr(*p*,*n*) or ^nat^Cr(*p*,*x*) reactions (^52^Cr: 83.8% natural abundance) by bombarding target material with low-energy protons (6<*E*_*p*_<20 MeV [41, 42]). Thus, ^52^Mn can be produced at medical centers using protons from a low-energy cyclotron without expensive isotopically enriched target material. So far, ^52^Mn has been used in several *ex vivo* and *in vivo* studies, including the biodistribution of Mn(II) [43], cell tracking [44], antibody imaging [39], and as a positron-emitting surrogate of a MEMRI contrast agent [34].

In this work, we utilized the radioisotope ^52^Mn to study the biodistribution of free Mn(II) in sixteen different tissues in mice following intravenous injection or inhalation in saline solution.

## Materials and methods

### Materials

Copper sheet (0.762 mm thick, 99.9% purity) was purchased from ESPI Metals (Ashland, Oregon, United States), and natural abundance chromium was electroplated by Four Star Finishing (Saint Louis, Missouri, United States). Nitrogen (N_2_) gas was purchased from Cee Kay Supply (Saint Louis), and all other chemicals were purchased from Sigma-Aldrich (Saint Louis), unless otherwise indicated.

### Production of ^52^Mn

Manganese-52 was produced from the ^nat^Cr(*p*,*x*) reaction using the CS-15 cyclotron at Washington University School of Medicine in Saint Louis as previously reported [41, 42]). We developed a method for fabricating batches of thin chromium foils that would fit into our target holders [41], similar to the one used by Tanaka and Furukawa [45]. Non-enriched chromium was electroplated onto copper metal sheet in an industrial “hard chrome” electroplating bath (Four Star Finishing, Saint Louis, United States). Following electroplating, the copper backing was removed by repeatedly digesting it in diluted nitric acid in a large glass pan, eventually leaving the electrodeposited layer of chromium as a batch of thin metal foils [41, 45]. In previously published results [41], the only contaminants >100 ppm (within uncertainty) were Sn (1195 ppm) and Se (134 ppm) in the final product. These elemental impurities were discovered by ICP-MS in a dissolved chromium foil that was never irradiated but was produced in a similar manner to the foils used in this work. For each production of ^52^Mn, one or more foils of this chromium metal was placed in a target holder and bombarded with protons from the CS-15 cyclotron (The Cyclotron Corporation, Berkeley, California, United States) [41]. Gamma-ray spectroscopy results from a non-dissolved chromium foil from a previous bombardment showed a radionuclidic purity of >99.5% (activity%) ^52^Mn (<0.5% ^54^Mn).

Several separation procedures have been published for ^52^Mn from a chromium metal target [39, 43, 46–52]. Following a method published by Buchholz, et al. in 2013 [50], cation-exchange chromatography was used to separate ^52^Mn from the chromium metal target after proton bombardment. The irradiated chromium metal was dissolved in diluted hydrochloric acid (for the biodistribution-only study: 5.5 mL of 1:10 (v/v) c. HCl:H_2_O). The solution was repeatedly evaporated and resuspended in sulfuric acid to replace the chloride anions with sulfate, then diluted to ~0.1 M sulfuric acid. Next, the solution was transferred to a simple gravity-flow column (inner diameter = 1.0 cm) consisting of AG 50W-X8 cation-exchange resin (Bio-Rad, Hercules, California, United States) that was pre-conditioned in 0.1 M sulfuric acid. The majority of the Cr(III) passed through the column and was further eluted in 0.1 M sulfuric acid, while ^52^Mn(II) was immobilized on the column. Of the ^52^Mn that was measured in the top chromium foil, >90.9% was eluted in 6 M HCl. The resulting solution was brought to dryness using heat and/or (N_2_) gas, and redissolved in saline. If necessary, solutions of hydrochloric acid, sodium hydroxide, and/or ammonium hydroxide were added to adjust the pH of the resuspended saline product to pH 4.0–5.0. When manganese is dissolved in HCl, it is expected to form manganese(II) chloride, which is soluble in water; manganese(IV) is much less soluble in water, and we did not observe any insolubility of our radiomanganese. For the inhalation study, the final solution that was placed inside the nebulizer was between 13.1–13.3 MBq (355–360 μCi) in 600 μL. The isotonicity of both the IV and inhaled doses was not evaluated in the current study, so it is possible that the concentrations of metal cation contaminants were great enough to influence the pharmacokinetics of the ^52^Mn(II). Metal impurities in our ^52^Mn will be addressed in future studies.

### Administration of ^52^Mn

All animals were purchased from Charles River Laboratories (Wilmington, Massachusetts, United States) and allowed to eat and drink *ad libitum*. All experiments involving animal subjects were performed under a protocol that was approved by the Animal Studies Committee of Washington University in St. Louis. Male CD-1 mice were used for biodistribution studies for IV injection and aerosol inhalation.

For the cohorts that received IV injection, mice (n = 4) were anesthetized by inhalation of isoflurane in a chamber before each mouse was removed and injected in the tail vein with ~100 μL volume, ~0.2–0.4 MBq (~5–10 μCi) of ^52^Mn. One extra syringe was prepared as a standard for later determining the count rate per volume of injectate using the gamma counter (see below). Each syringe, including the standard syringe, was weighed pre- and post-injection to determine the mass of injectate that was actually expelled.

In the inhalation studies, doses of ^52^Mn salt solutions were administered to mice as previously reported by our research group [53, 54] for other radiolabeled compounds. Solutions were loaded into a Small Volumetric Mean Diameter (VMD) Nebulizer Unit (Aeroneb Lab, Aerogen, Dangan, Galway, Ireland) with the following specifications: >0.1 mL·min^-1^ flow rate, 4.0–6.0 μm VMD, <0.2 mL residual volume. The exit port of the nebulizer was inserted into a clear plastic chamber (20.3×20.3×11.4 cm l×w×h). Four mice for each timepoint were inserted individually, without any anesthesia, into four tubes with a plastic plunger to gently push the body to the front of the tube and snout through a small opening at the opposite end of the tube. The ^52^Mn solution was pipetted into the liquid reservoir of the nebulizer and then the nebulizer was powered on, producing an aerosol that filled the chamber. The four mice were allowed to inhale the aerosol for ~4–7 min., then the tubes were detached, and the mice were removed. One of the mice from each inhalation group of four mice was euthanized immediately following removal from its tube to serve as the standard “dose mouse” for the dose received by that group, leaving three mice alive from each administration by inhalation.

### Ex vivo biodistribution

At each timepoint post-administration (by injection or inhalation), animals were euthanized, dissected, and then each tissue was weighed and analyzed for radioactivity using a sodium iodide gamma-ray counter (Beckman 8000, Beckman Instruments, Irvine, California, United States). For the injection study, the injectate from the standard syringe was diluted, and one unique aliquot of this dilution was assayed after the tissues from each animal. For the inhalation study, the whole carcass of the “dose mouse” from each inhalation group was assayed once by the gamma counter. For the samples from each administration route, a single background measurement was performed, and the count rate (counts per minute, CPM) from each sample of tissue (or of diluted standard) was corrected by background subtraction and by decay correction (neglecting decay during gamma counting). The corrected CPM from the diluted standard was used to estimate the (corrected) CPM from the same mass of undiluted standard. The corrected CPM from each tissue sample was normalized both to the mass of the tissue sample (in grams, g) and to the estimated CPM from the mass of undiluted injectate that was actually delivered to the corresponding animal (injected dose, ID). Thus, the relative concentration of ^52^Mn in each tissue sample was calculated as %ID/g.

### Statistical and uncertainty analysis

For each tissue and timepoint, results were averaged across animals—n = 4 in the injection study and n = 3 in the inhalation study—and statistical analysis was performed. The uncertainty in the final result for each tissue reflects only sample standard deviation across animals for that tissue. Statistical significance between the biodistribution at 1 d after injection or inhalation was determined using Welch’s *t*-test, which is a correction to Student’s *t*-test that is applied when performing a hypothesis test on sample means from two unpaired samples of different size and variance, as was the case in this comparison. This test was administered using the TTEST function in *Microsoft Excel for Mac* (various versions, Microsoft, Redmond, Washington, United States) for a two-tailed *t*-test between two samples of heteroscedastic (unequal variance) data.

## Results

**Fig 1:**
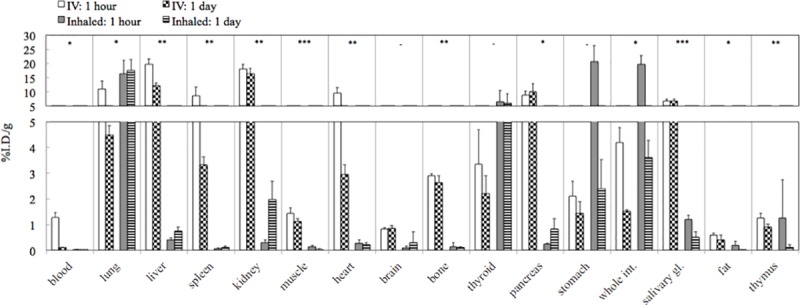
Plot showing results from *ex vivo* biodistribution of saline solutions containing ^52^Mn administered via intravenous injection or inhalation. The timepoints shown represent the time after administration of the dose. Results for the *p*-values from Student’s *t*-test with Welch’s correction at 1 d p.i. only: *: *p*<5%; **: *p*<0.1%; ***: *p*<0.01%; -: *p*≥5%. Sample sizes for each timepoint were n = 4 mice for injection and n = 3 for inhalation. Uncertainties: one absolute standard deviation in units of %ID/g. Error bars were only drawn in the positive direction for visual clarity. The plotted data is presented in numerical form in Table 1.

For the biodistribution-only study, bombardment of ^nat^Cr metal foils with ~13.4 MeV protons for a total of 27 μA·h (12 μA), which produced 41 MBq (1.1 mCi) (decay-corrected to end-of-bombardment), which was 69.6% of theoretical yield based on our published cross-section results [41]. Fig 1 and Table 1 show the results from *ex vivo* biodistribution of ^52^Mn in saline administered via intravenous injection or by inhalation. Manganese-52 was cleared rapidly from the blood, as demonstrated by its rapid decrease from 1 h to 1 d p.i., and the activity in the gastrointestinal tract—stomach and intestines—also decreased rapidly between these timepoints. At 1 h p.i., the highest activity was found, in decreasing order, in the liver, kidney, lung, heart, pancreas, spleen, and salivary glands (Table 1); however, by 1 d p.i., the concentration of ^52^Mn in all of these organs of high initial uptake had decreased substantially, except for the salivary glands, pancreas, and kidney, as shown in Fig 1. Among the other tissues, which had lower uptake at 1 h p.i., ^52^Mn was retained to various degrees in the brain, bone, and thyroid.

**Table 1:** Results from *ex vivo* biodistribution of saline solutions containing ^52^Mn administered via intravenous injection or inhalation.

	Uptake of ^52^Mn (%ID/g)				*t*-test (*p*)
	Intravenous		Inhalation		
	1 h	1 d	1 h	1 d	1 d
Blood	1.3 ± 0.2	0.10±0.02	0.019±0.008	0.007±0.003	0.0011
Lung	11 ± 3	4.5±0.4	16±5	18±4	0.026
Liver	20 ± 2	12±1	0.39±0.09	0.7±0.2	0.00013
Spleen	9 ± 3	3.3±0.3	0.07±0.03	0.12±0.05	0.00025
Kidney	18 ± 2	17±2	0.31±0.09	2.0±0.7	0.00012
Muscle	1.4 ± 0.2	1.13±0.09	0.14±0.05	0.04±0.03	0.000028
Heart	10 ± 2	2.9±0.4	0.3±0.1	0.23±0.08	0.020
Brain	0.8±0.1	0.9±0.1	0.09±0.07	0.3±0.4	0.00034
Bone	2.90±0.08	2.6±0.3	0.1±0.2	0.11±0.03	0.14
Thyroid	3±1	2.2±0.7	6±4	6±3	0.00026
Pancreas	9±1	10±3	0.25±0.02	0.8±0.4	0.17
Stomach	2.1±0.6	1.4±0.5	21±6	2±1	0.0073
Whole Intestine	4.2±0.6	1.51±0.06	20±3	3.6±0.7	0.00029
Salivary Gland	6.7±0.8	6.8±0.6	1.2±0.2	0.5±0.2	0.29
Fat	0.58±0.09	0.4±0.2	0.2±0.2	0.004±0.001	0.031
Thymus	1.3±0.2	0.9±0.1	1±1	0.1±0.1	0.000074

The timepoints shown represent time after administration of the dose. Sample sizes for each timepoint were n = 4 mice for injection and n = 3 for inhalation. Uncertainties: one absolute standard deviation in units of %ID/g (i.e., percentage does not imply relative sample standard deviation). All values for uncertainty were rounded to one significant digit, and the mean value was rounded to a matching number of decimal places. Results for the *p*-values from Student’s *t*-test with Welch’s correction are shown rounded (up or down) to two significant digits. The numerical results in this table have been plotted in Fig 1.

Comparing the biodistribution of ^52^Mn(II) at 1 d after IV injection or inhalation, we considered two sample means to be statistically significant if the *p*-value from the *t*-test was <0.05. Applying the *t*-test and this cutoff value, nearly all tissues that we measured showed a statistically significant difference, with the exception of brain, thyroid, and stomach, all of which demonstrated large relative sample standard deviations (%RSD). Salivary glands and muscles demonstrated especially strong statistical significance (*p*<0.0001). S1 Fig (Supplementary Information) shows co-registered PET/CT images of two mice (species: C57-Black-6) from a separate but similar study in which larger doses of [^52^Mn]MnCl_2_ were administered for *in vivo* PET imaging. These images demonstrate a distribution of PET signal in many of the same tissues with high uptake in the current *ex vivo* biodistribution results, and these images suggest the potential utility of ^52^Mn for further quantitative PET/CT studies.

## Discussion

For the comparison of IV injection to inhalation of ^52^Mn in mice, the concentration of ^52^Mn in most tissues at both 1 h and 1 d timepoints is higher than in the inhalation results. The activity in the gastrointestinal tract was much greater in the inhalation results, suggesting that some of the dose may have been swallowed directly from the aerosol or inhaled, cleared by mucociliary clearance, and then swallowed. Also in our studies, we observed uptake in the pancreas and brain, while ^52^Mn that entered the stomach or intestines did not appear to be retained, although the one hour amounts were much higher in the inhalation groups likely due to putative swallowing of some of the dose. Retention of ^52^Mn in the brain, thyroid, and thymus was observed in results from both injection and inhalation. We also found significant uptake of ^52^Mn at 1 d in the bone (2.6%ID/g) resulting from injection, but not from inhalation.

For both injection and inhalation, ^52^Mn was cleared rapidly from the blood, and the injection data showed ^52^Mn in the liver and kidney in <1 h. The inhalation data also suggest accumulation in the liver and kidneys, but this accumulation took much longer than one hour. Although manganese appears to accumulate in both liver and kidneys, results from the literature [22–25] suggest that very little manganese is excreted in urine. Instead, the vast majority of manganese is excreted in feces, primarily due to release of manganese from the liver into the enterohepatic circulation. In other studies, animals that were administered manganese or radiomanganese intravenously [17, 22, 55–58], high uptake was generally observed in the kidney, pancreas, liver, adrenal glands, and intestine, as well as comparatively small amounts in the brain. For intratracheal instillation, Heilig, et al. [59] and Brain, et al. [60] reported that [^54^Mn]MnCl_2_ was retained in the lungs to large extent, but was also found in significant amounts in the intestines, liver, kidneys, and in small amounts in the brain. These intratracheal instillation studies also showed that Mn can enter the bloodstream from the lungs, possibly by ion channels, but not by the divalent metal transporter-1 (DMT-1) in that particular tissue [59, 60]. These general trends in manganese distribution agree with studies that focused more narrowly on the *in vitro* or *in vivo* uptake of manganese by cells or tissues that included liver [25, 61], kidney [61], pancreas [61], intestines [60, 62], salivary glands [17, 63], myocardium [58], and bone [64].

Owing to its neurotoxic effects, particular interest has been paid to uptake of manganese in the brain by various routes. Kanayama, et al. [65] administered to mice by eight different routes a solution containing sixteen different radiotracers, mostly transition metals, and, for most administration routes, ^54^Mn(II) had higher brain uptake than most of the other tracers. In several other studies, rodents received radiomanganese by carotid injection or continuous *in situ* brain perfusion brain. These studies suggest that manganese enters the brain by more than one mechanism, including carrier-mediated uptake of Mn-citrate [66], by store-operated calcium channels as Mn(II) [67], by transferrin-receptor mediated endocytosis as Mn-transferrin [68, 69], or by other unspecified mechanism(s) that are faster than simple diffusion [69, 70]. In a comparison of ^54^Mn species administered by *in situ* brain perfusion, Mn-citrate was transported across the blood-brain barrier faster than either free Mn(II) or Mn-transferrin, suggesting that Mn-citrate might be an important route for manganese uptake in the brain [66]. Furthermore, it is likely that manganese accumulates in the brain because it effluxes at a rate that is consistent with simple diffusion [71, 72].

Since certain inhaled substances can enter the brain directly from the olfactory bulb—without entering the bloodstream—there have been a considerable number of studies examining the possibility of manganese entering the brain by such mechanisms. Characterization of this mechanism(s) would be particularly relevant to the problem of inhaled manganese in metal workers. Animal studies using radiomanganese [73, 74] or MEMRI [75, 76], suggested that Mn(II) can be transported from the olfactory bulb and into olfactory neurons, transported through those neurons, across synaptic junctions, into secondary olfactory neurons, and then into the diencephalon and cerebrum, and (in rats) into the spinal cord. Thompson, et al. [77] tested uptake in rats with defective divalent metal transporter-1 (DMT-1) proteins and confirmed that this transporter is involved in manganese uptake in the rat olfactory bulb based on higher uptake in healthy control rats. In rats, ninety minute nose-only inhalation of aerosolized [^54^Mn]MnCl_2_ [78] or [^54^Mn]MnHPO_4_ [79] solution leads to uptake in the olfactory bulb and olfactory tubercle. In both studies, they observed uptake in lungs, liver, kidney, and pancreas, and—at much lower amounts—in the striatum of the brain. In a similar study, Lewis, et al. [38] exposed mice and rats to nebulized, non-radioactive MnCl_2_ for 10 nose-only doses of 6 hours each. Sample analysis by proton induced X-ray emission (PIXE) revealed elevated manganese in trigeminal ganglia, suggesting this nerve as a possible pathway for brain entry of manganese. Along with these published examples of brain uptake of Mn cations, the present study has shown uptake of ^52^Mn(II) in the mouse brain following administration by IV injection or inhalation or aerosol. The magnitude of uptake in the brain that we observed was low compared to some other tissues, with uptake being greater following IV injection than from inhalation. However, the presence of an exogenous substance in the brain in any amount demonstrates an ability to cross the blood-brain barrier, which is an important result in itself.

In our studies, relatively high uptake was observed in the lung and thyroid following either route of administration. Although several papers confirmed thyroid uptake of manganese from different administration routes in animal models [80–83], the biology of uptake of manganese in the thyroid does not seem clear [83]. Interestingly, electron spin resonance (ESR) has shown that only a small fraction of the manganese naturally found in the rat thyroid is in the 2+ oxidation state [37], perhaps suggesting that it is bound to serum protein(s) as Mn(III) and not present as a free Mn(II). Additionally, following subcutaneous injection of Mn(II)Cl_2,_ in guinea pigs [81], manganese rapidly accumulated in thyroid by 1 d p.i., but then 70% of the manganese in the thyroid at 1 d had cleared by 2 d p.i.; and then 85%, by 96 hours p.i. Inhaled manganese uptake in salivary glands was in agreement with observations in welders exposed to manganese [84]. Interestingly, our results showed that injection of [^52^Mn]MnCl_2_ exhibited higher and faster uptake in salivary gland than in the inhalation study. Our results demonstrate uptake of ^52^Mn in the brain, thyroid, and pancreas in all trials. Interestingly, uptake from IV administration was higher in brain and pancreas, but lower in thyroid, compared to inhalation results. Importantly, Graves, et al. [85] have shown that anesthesia by isoflurane can significantly decrease uptake of Mn(II) in the pancreas in fasted mice. This study concluded that isoflurane initiates a sequence in pancreatic *β*-cells that reduces opening of voltage-dependent calcium channels (VDCCs)—the channels through which Ca(II), Mn(II), and potentially other divalent cations can enter the pancreatic beta cells. In one part of this work, ^52^Mn(II) in aqueous sodium acetate was administered IV with and without anesthesia by isoflurane, and the uptake in the pancreas at 1 h p.i. under anesthesia was similar to our result (in %ID/g). However, the uptake in the pancreas without anesthesia was ~3x greater than with anesthesia. Since our study only used anesthesia during IV administration of ^52^Mn(II), and not during inhalation, we expect that in the absence of anesthesia in both routes of administration the difference would be even greater in uptake in the pancreas between IV injection and inhalation.

Along with recently renewed interest in manganese for signal in both MRI and PET, Brunnquell, et al. [40] have published results for brain uptake of manganese in female rats following tail vein infusion at 2 mL/h of non-carrier-added ^52^Mn(II) in radiotracer concentrations. In addition to monitoring brain uptake of ^52^Mn(II), this study included results for biodistribution from several tissues at 4 and 48 h p.i. Compared to Brunnquell, et al., our biodistribution results were roughly 10x greater for many tissues (in %ID/g), likely due to a roughly 10x lower body mass of the mice in our study compared to rats, along with different timepoints and gender. However, the relative changes from 4 to 48 h p.i. in this study was similar to our results at 1 h and 1 d, including brain, spleen, liver, intestine, heart, lung, and muscle, while results for blood, pancreas, thymus, kidney, and bone demonstrated results that were less similar to our results, based on tissue concentrations relative to other tissues and/or relative changes from 4 to 48 h. Manganese-52 might someday be used as a PET agent for niche clinical research applications, but our biodistribution results in mice can be used, in conjunction with other published results, to inform regulations for safe levels of environmental and occupational exposure to manganese. The primary objective of this work was to contribute to basic research with regards to the *in vivo* behavior of manganese cations, which are increasingly being studied preclinically for PET agents and MRI contrast agents—with only the possibility for future translation to the clinic.

## Conclusion

In this work, we have produced ^52^Mn via the ^nat^Cr(*p*,*x*) reaction, chemically isolated this radioisotope of manganese from the chromium metal target material, and reported new data for the biodistribution of manganese in mice following administration by IV injection or inhalation. Manganese-52 was produced by cyclotron bombardment with low-energy protons on chromium metal, and subsequent chemical separation of ^52^Mn(II) was performed by cation-exchange chromatography and re-dissolved in saline solution for *ex vivo* biodistribution studies in mice. The doses were administered by IV injection or inhalation, always resulting in uptake of ^52^Mn in the brain, thyroid, and pancreas. Interestingly, uptake from IV administration was higher in brain and pancreas, but lower in thyroid, compared to inhalation results. While ^52^Mn might find use as a PET agent in preclinical studies, our biodistribution results in mice may be used to inform regulations for safe levels of environmental and occupational exposure to Mn(II) and the predicted biodistribution of Mn(II) following exposure by IV injection or inhalation.

## Disclaimer

“This report was prepared as an account of work sponsored by an agency of the United States Government. Neither the United States Government nor any agency thereof, nor any of their employees, makes any warranty, express or limited, or assumes any legal liability or responsibility for the accuracy, completeness, or usefulness of any information, apparatus, product, or process disclosed, or represents that its use would not infringe privately owned rights. Reference herein to any specific commercial product, process, or service by trade name, trademark, manufacturer, or otherwise does not necessarily constitute or imply its endorsement, recommendation, or favoring by the United States Government or any agency thereof. The views and opinions of authors expressed herein do not necessarily state or reflect those of the United States Government or any agency thereof.”

## Supporting information

S1 Fig PET/CT images and post-imaging, ex vivo biodistribution for two mice following intravenous administration of ^52^Mn in aqueous solution. At 1 h p.i., ^52^Mn was observed in the digestive tract, kidneys, and likely the pancreas. At 3 d p.i., ^52^Mn had cleared from the digestive tract, while it is retained in the kidneys, as well as liver, pancreas, and thyroid gland. Manganese-52 was eluted from the cation-exchange column in 0.067 M ammonium oxalate solution. The ammonium oxalate product was heated to dryness, and then the heat was increased to burn away the ammonium oxalate. Manganese-52 was resuspended by adding water followed by a drop of 6 M hydrochloric acid, then heated to dryness. Evaporation and resuspension in water was repeated and then the ^52^Mn was finally resuspended in water, resulting in a solution with pH of ~6.5. Mice (n = 2; C57-Black-6; male) were anesthetized by isoflurane (1–2% induction), injected in the tail vein with ~41 μCi of ^52^Mn in 50 μL total volume, and imaged simultaneously side-by-side at 1 h and 3 d p.i. At the imaging timepoints, anatomic images were obtained by non-contrast CT using an Inveon small animal PET/CT scanner (Siemens Preclinical Solutions, Knoxville, Tennessee, United States), and PET data were acquired using either the Inveon PET/CT scanner or a microPET Focus 220 scanner (Siemens Preclinical Solutions). Static PET data were acquired for 30 minutes at the 1-hour timepoint and for 1 hour at the 3-day timepoint. Attenuation maps for each subject were obtained from either the CT scan in the Inveon scanner or by a transmission scan on the Focus 220 scanner. Images were analyzed using *Inveon Research Workplace* software (Siemens Preclinical Solutions).
(TIF)

S1 File Data analysis of the cohorts of mice that received [^52^Mn]MnCl_2_ via IV injection. This file is a workbook made using *Microsoft Excel for Mac* and contains multiple sheets.
(XLSX)

S2 File Data analysis of the cohorts of mice that received [^52^Mn]MnCl_2_ via inhalation. This file is a workbook made using *Microsoft Excel for Mac* and contains multiple sheets.
(XLSX)

S3 File Comparison of results from IV injection and inhalation of [^52^Mn]MnCl_2_. This file is a workbook made using *Microsoft Excel for Mac* and contains multiple sheets. The workbook includes results from S1 File and S2 File, comparison of these results by plotting, and hypothesis testing of these results by Student’s *t*-test with Welch’s correction at 1 d p.i. only.
(XLSX)
