# Supplementary figures and images for: Biodistribution and PET Imaging of pharmacokinetics of manganese in mice using Manganese-52
Source: PLoS One. 2017 Mar 17;12(3):e0174351. doi: 10.1371/journal.pone.0174351 (PMC5357058; doi:10.1371/journal.pone.0174351)

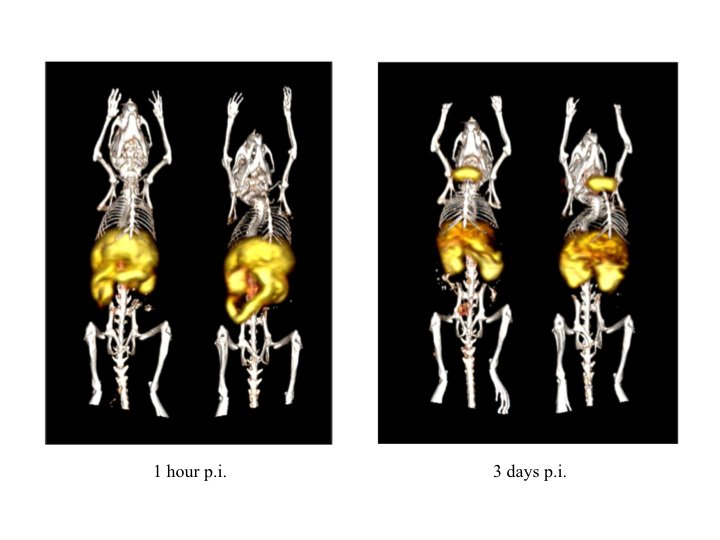

Supplement: S1 Fig — At 1 h p.i., 52Mn was observed in the digestive tract, kidneys, and likely the pancreas. At 3 d p.i., 52Mn had cleared from the digestive tract, while it is retained in the kidneys, as well as liver, pancreas, and thyroid gland. Manganese-52 was eluted from the cation-exchange column in 0.067 M ammonium oxalate solution. The ammonium oxalate product was heated to dryness, and then the heat was increased to burn away the ammonium oxalate. Manganese-52 was resuspended by adding water followed by a drop of 6 M hydrochloric acid, then heated to dryness. Evaporation and resuspension in water was repeated and then the 52Mn was finally resuspended in water, resulting in a solution with pH of ~6.5. Mice (n = 2; C57-Black-6; male) were anesthetized by isoflurane (1–2% induction), injected in the tail vein with ~41 μCi of 52Mn in 50 μL total volume, and imaged simultaneously side-by-side at 1 h and 3 d p.i. At the imaging timepoints, anatomic images were obtained by non-contrast CT using an Inveon small animal PET/CT scanner (Siemens Preclinical Solutions, Knoxville, Tennessee, United States), and PET data were acquired using either the Inveon PET/CT scanner or a microPET Focus 220 scanner (Siemens Preclinical Solutions). Static PET data were acquired for 30 minutes at the 1-hour timepoint and for 1 hour at the 3-day timepoint. Attenuation maps for each subject were obtained from either the CT scan in the Inveon scanner or by a transmission scan on the Focus 220 scanner. Images were analyzed using Inveon Research Workplace software (Siemens Preclinical Solutions). (TIF) [file pone.0174351.s001.tif]
